# Exploring cryptic amyloidogenic regions in prion-like proteins from plants

**DOI:** 10.3389/fpls.2022.1060410

**Published:** 2023-01-16

**Authors:** Carlos Pintado-Grima, Jaime Santos, Valentín Iglesias, Zoe Manglano-Artuñedo, Irantzu Pallarès, Salvador Ventura

**Affiliations:** ^1^ Departament de Bioquímica i Biologia Molecular, Institut de Biotecnologia i Biomedicina, Universitat Autònoma de Barcelona, Barcelona, Spain; ^2^ Barcelona Institute for Global Health, Barcelona Centre for International Health Research (ISGlobal, Hospital Clínic-Universitat de Barcelona), Barcelona, Spain; ^3^ Nanomalaria Group, Institute for Bioengineering of Catalonia (IBEC), The Barcelona Institute of Science and Technology, Barcelona, Spain

**Keywords:** cryptic amyloidogenic regions, prion-like domains, plants, functional interactions, bioinformatics

## Abstract

Prion-like domains (PrLDs) are intrinsically disordered regions (IDRs) of low sequence complexity with a similar composition to yeast prion domains. PrLDs-containing proteins have been involved in different organisms’ regulatory processes. Regions of moderate amyloid propensity within IDRs have been shown to assemble autonomously into amyloid fibrils. These sequences tend to be rich in polar amino acids and often escape from the detection of classical bioinformatics screenings that look for highly aggregation-prone hydrophobic sequence stretches. We defined them as cryptic amyloidogenic regions (CARs) and recently developed an integrated database that collects thousands of predicted CARs in IDRs. CARs seem to be evolutionary conserved among disordered regions because of their potential to stablish functional contacts with other biomolecules. Here we have focused on identifying and characterizing CARs in prion-like proteins (pCARs) from plants, a lineage that has been poorly studied in comparison with other prionomes. We confirmed the intrinsic amyloid potential for a selected pCAR from *Arabidopsis thaliana* and explored functional enrichments and compositional bias of pCARs in plant prion-like proteins.

## 1 Introduction

Since the description of the first self-perpetuating proteinaceous infectious pathogens in the late 20^th^ century (Prusiner, 1982) and the subsequent discovery of yeast prions (Wickner, 1994), the prion concept has progressively evolved with our understanding of the phenomenon. Protein domains in a variety of polypeptides were found to share similar sequential composition to the domains required for yeast prion conversion, although they do not necessarily show conformational transitions or mechanisms of self-propagation and were termed prion-like domains (PrLDs). Despite initially thought to be linked to disease, further characterization of prion-like polypeptides revealed their role in regulating key biological functions in a wide variety of organisms (True and Lindquist, 2000; Chakrabortee et al., 2016; Yuan and Hochschild, 2017; Pallares et al., 2018; Tetz and Tetz, 2018). In this context, under physiological conditions, most PrLDs-containing proteins would not transition towards aggregated states, and instead, they tend to be involved in functional interactions with other proteins and nucleic acids (Alberti et al., 2009; King et al., 2012; Iglesias et al., 2015).

PrLDs are often enriched in glutamine, asparagine, serine, glycine, and tyrosine (Michelitsch and Weissman, 2000; Alberti et al., 2009; Toombs et al., 2010; Diaz-Caballero et al., 2018) and depleted of hydrophobic amino acids, thus having an overall polar character. The characteristic compositional bias of PrLDs is at the core of diverse predictive bioinformatics tools that aim at identifying such signatures in protein sequences (Toombs et al., 2012; Lancaster et al., 2014; Afsar Minhas et al., 2017). Most of the current protein aggregation predictors were designed to predict highly hydrophobic sequence stretches in disease-associated proteins, a feature that does not match the polar nature of PrLDs (Fernandez-Escamilla et al., 2004; Conchillo-Sole et al., 2007; Garbuzynskiy et al., 2010; Maurer-Stroh et al., 2010; Sormanni et al., 2015). Only a few of them, like pWaltz (Sabate et al., 2015b), and its implementation in PrionW (Zambrano et al., 2015), use adapted aggregation scales aimed to identify sequences of milder amyloid propensity, denominated soft amyloid cores (SACs). Albeit pWaltz has been successfully used to identify SACs within PrLDs whose correspondent peptides have been experimentally validated to form amyloids (Sant’anna et al., 2016; Batlle et al., 2017a), this algorithm suffers from technical limitations, including the exclusion of proline residues on its predictions, the identification of a single hit per PrLD or the rigidity of its sequence scoring system that unavoidably employs a sliding window of 21 residues.

In recent work, we implemented a pWaltz-related approach to identify protein regions with amyloid potential in hydrophilic sequential contexts by decreasing the actual detection threshold of the Walz algorithm (Maurer-Stroh et al., 2010), -a well-characterized amyloidogenic predictor- without any further arbitrary residue exclusion or window-size length assumption. This prediction scheme allowed us to identify and characterize cryptic amyloidogenic regions (CARs) in experimentally validated intrinsically disordered regions (IDRs), which were previously considered to be depleted of aggregation-prone regions (Santos et al., 2021). CARs are sequences of mild amyloidogenic potential, yet able to assemble into fibrils when in isolation. These stretches have likely been conserved within IDPs because their potential to act as functional interacting motifs with other biomolecules overpasses a low risk for pathogenic aggregation. In fact, CARs consistently overlap with interacting motifs in IDPs. We collected these predictions in a comprehensive database named CARs-DB (Pintado-Grima et al., 2022a).

Given the generic disordered nature of PrLDs, in this work, we conducted a computational screening to identify CARs in plant PrLDs (pCARs) from different plant species to understand their role in this kingdom. We discovered that CARs are widespread in model plants’ PrLDs, with gene ontology enrichment analysis suggesting they are connected to critical biological processes. In addition, we validated amyloid formation for a selected pCAR from *Arabidopsis thaliana* (*A. thaliana*), demonstrating that this class of plant protein sequences bears the potential to spontaneously self-assemble, at least *in vitro*.

## 2 Materials and methods

### 2.1 Data collection and dataset generation

The reference proteomes of five different plant model organisms, including *Arabidopsis thaliana* (mouse-ear cress; UP000006548), *Zea mays* (maize; UP000007305), *Oriza sativa* (rice; UP000059680), *Solanum lycopersicum* (tomato; UP000004994) and *Nicotiana tabacum* (tobacco plant; UP000084051) were extracted from UniProt (UniProt, 2021) (Release 2022_03). PrLDs were screened with PLAAC (Lancaster et al., 2014) using a core length of 60 amino acids and relative weighting of background probabilities obtained from input sequences. PLAAC searches protein sequences to identify probable prion subsequences exploiting a hidden-Markov model (HMM) algorithm using the selected background probabilities. pCARs longer than six residues were obtained from PrLDs by applying the Waltz algorithm at thresholds 73.5, 80, and 85, as optimized in our original study of CARs (Santos et al., 2021) and CARs-DB (Pintado-Grima et al., 2022a) to account for polar amyloidogenic regions. The Waltz algorithm (Maurer-Stroh et al., 2010) explores the sequence determinants of amyloid structure using position-specific scoring matrices derived from analyzing a database of short amyloid-forming and non-forming sequences. Modified Waltz thresholds optimized in our original CAR work were selected based on previous experimental evidence (Sabate et al., 2015b).

### 2.2 Gene Ontology (GO) enrichment analysis

Functional enrichments were assessed with the database for annotation, visualization, and integrated discovery DAVID (Sherman et al., 2022) using each proteome as background. GO terms of biological process (BP_DIRECT), molecular function (MF_DIRECT), and cellular component (CC_DIRECT) were selected for the study with a modified Fisher cutoff p-value of 0.1. The Bejamini false discovery rate method was used to control increased error rates for multiple tests (Benjamini and Hochberg, 1995).

### 2.3 Peptide preparation and aggregation

The peptide encoding the mediator of RNA polymerase II transcription subunit 9 (MED9) pCAR region Ac-QYQQFQQQQHFIQQQQFQ-NH_2_ was purchased from SynPeptide (Shanghai, China) with a purity >95%. To mimic the protein environment, terminal charges were neutralized by N-terminal acetylation and C-terminal amidation. Peptide powder was dissolved in hexafluoroisopropanol to a concentration of 1 mg/mL, aliquoted, and vacuum dried with a SpeedVac (Thermo Fisher Scientific, Waltham, USA). For the aggregation reactions, aliquots were solubilized with 20 µL hexafluoroisopropanol and diluted to a concentration of 50 μM in 20 mM Tris and 100 mM NaCl pH 8. 150 μL of peptide solutions were incubated in a 96 wells plate (non-treated) (Sarstedt, Germany) for two days at 37°C with continuous agitation at 100 RPM. A detailed description of the aggregation conditions is described in the supplementary MIRRAGGE spreadsheet (Martins et al., 2020).

### 2.4 Binding to amyloid dyes

End-point samples of the MED9 pCAR were incubated with 40 µM thioflavin-T (Th-T) for 20 minutes, and their fluorescence emission spectra were recorded using a Spark plate reader (Tecan, Männedorf, Switzerland). Fluorescence intensity was measured by exciting at 440 nm, bandwidth of 5 nm, and collecting the emission from 460 to 600 nm with a 1 nm interval.

Congo-red (CR) binding to end-point samples recovered from the plate was tested using a Cary 100 UV/Vis Spectrophotometer (Varian, Palo Alto, United States). 100 µl of the aggregated sample was incubated for 20 minutes with 900 µl of CR at a final concentration of 5 µM before recording the spectra. CR absorbance spectra were recorded from 400 to 700 nm. A control sample with 100 µl of the aggregation buffer was prepared (CR-Free).

### 2.5 Far circular dichroism spectroscopy

Far-UV circular dichroism (CD) spectra of the peptide before and after aggregation were recorded on a Jasco J-815CD spectrometer (Halifax, Canada) at 25°C. The spectra were acquired from 260 nm to 200 nm at 0.2 nm intervals, 2 nm bandwidth, 2 s of response time, and a 200 nm/min scan speed on a 0.1 cm quartz cell. To prevent unwanted self-association through the experiment and have a representative spectrum of the initial time-point, the initial aliquot was resuspended in 25 mM Sodium acetate 100 mM NaCl pH 5; 10 accumulations were recorded and averaged for each measurement.

### 2.6 Attenuated total reflectance Fourier transform infrared spectroscopy

Attenuated total reflectance Fourier transform infrared (ATR-FTIR) spectroscopy experiments were performed in a Bruker Tensor 27 FTIR spectrometer (Bruker Optics Inc) with a Golden Gate MKII ATR accessory. Samples were dried under an N_2_ (g) stream and measured at a spectral resolution of 2 cm^-1^ within the 1800–1500 cm^-1^ range (16 accumulations). Dara recording and normalization were performed using the OPUS MIR Tensor 27 software and deconvoluted with the Peak Fit 4.12 program (Systat Software Inc., San Jose, CA, USA).

### 2.7 Transmission electron microscopy

5 µL of the aggregated sample, previously diluted to a concentration of 5 µM, was placed onto glow-discharged carbon-coated copper grids for 1 min. Sample excess was blotted with ashless filter paper, and grids were washed in distillate water drops. Negative staining was performed with 2% (w/v) uranyl acetate for 1 min. A TEM JEM-1400 (JEOL, Peabody, USA) microscope was used, operating at an accelerating voltage of 120 kV.

## 3 Results

### 3.1 Prevalence of pCARs in plant PrLDs

**Figure 1:**
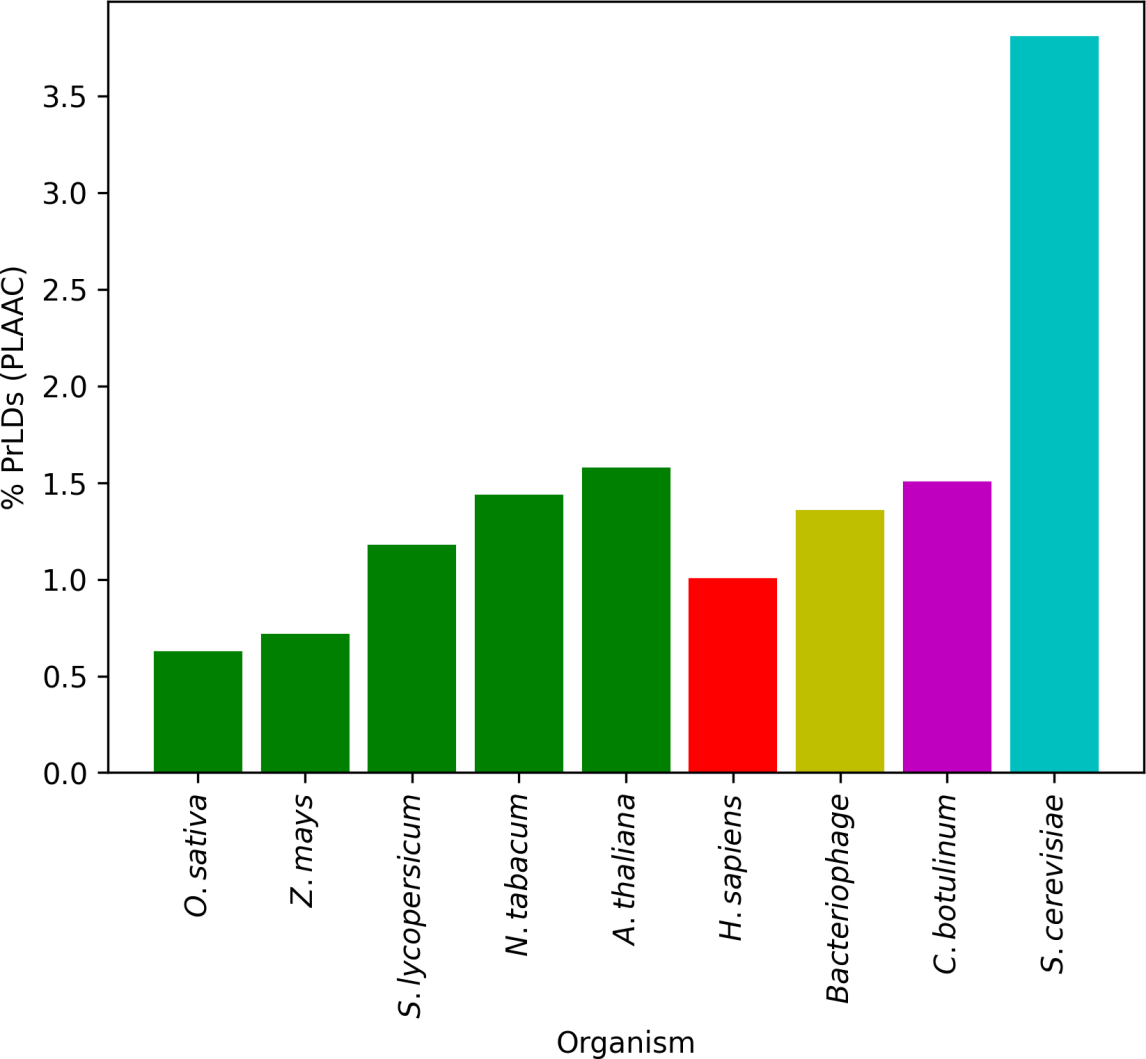
Proportion of predicted PrLDs for five models of plants in comparison with other described organisms. Around 1% of the total proteome is predicted as prion-like, similar to the values obtained for humans, bacteria, and viruses. *S. cerevisiae* represents an exceptional case of a proteome highly enriched in PrLDs.

Since the original characterization of prion domains in yeast (Chernoff et al., 1995; Serio et al., 1999), PrLDs with similar features have been identified and studied in other organisms such as viruses (Tetz and Tetz, 2018), bacteria (Iglesias et al., 2015; Yuan and Hochschild, 2017; Choi et al., 2022), protozoan (Pallares et al., 2018) or humans (An and Harrison, 2016; Iglesias et al., 2019). Plants have received less attention, with only a few studies focusing on PrLDs’ coverage and function (Chakrabortee et al., 2016; Dorone et al., 2021; Garai et al., 2021). To identify the potential presence of CARs in PrLDs (pCARs) from plants, the proteomes of five different model higher plants were first screened with the PLAAC algorithm. Around 1% of the total proteome of these species was predicted as PrLDs, spanning from 0.62% (*O. sativa*) to 1.57% (*A. thaliana*). In comparison with other studied model organisms (Gil-Garcia et al., 2021), this average is similar to the proportion of human, bacteria, and viruses PrLDs and lower than *S. cerevisiae* (~3%) (**Figure 1**; **Table 1**).

**Table 1:** Identification of pCARs from PrLDs for the five different plant model organisms.

Organism	Proteins	PrLDs	PrLDs (%)	Waltz threshold	CARs	PrLDs CAR+	PrLDs CAR+ (%)	CARs/PrLD CAR+	Average CAR length
*Arabidopsis thaliana*	27474	432	1.57	85	679	303	70.14	2.24	11.6
				80	983	356	82.41	2.76	12.66
				73.5	1484	402	93.06	3.69	14.69
*Zea mays* (maize)	56926	404	0.71	85	667	302	74.75	2.21	12.58
				80	918	356	88.12	2.58	13.78
				73.5	1358	380	94.06	3.57	15.99
*Oryza sativa* (rice)	43672	270	0.62	85	429	190	70.37	2.26	11.48
				80	595	220	81.48	2.7	13.19
				73.5	864	253	93.7	3.42	15.52
*Solanum lycopersicum* (tomato)	34655	405	1.17	85	689	307	75.8	2.24	13
				80	957	354	87.41	2.7	14.2
				73.5	1366	377	93.09	3.62	15.88
*Nicotiana tabacum* (tobacco plant)	61673	883	1.43	85	1471	683	77.35	2.15	12.38
				80	1989	763	86.41	2.61	13.98
				73.5	2913	847	95.92	3.44	15.96

PrLDs were screened with PLAAC, and their corresponding CARs were predicted with Waltz at the three designated thresholds.

**Figure 2:**
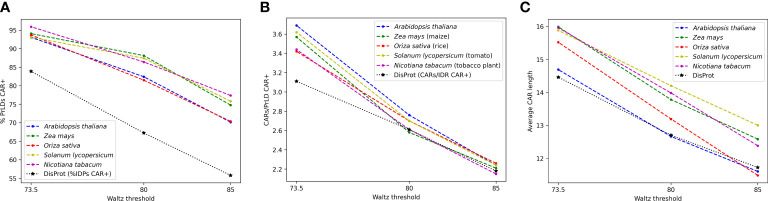
General statistics for pCARs from *A thaliana* (blue), *Z. mays* (green), *O. sativa* (red), *S. lycopersicum* (ochre), and *N. tabacum* (purple), in comparison with general DisProt CARs (black). Most PrLDs are CAR-positive **(A)**, often with more than one predicted pCARs **(B)**. Lower Waltz thresholds contain more pCARs with longer average sequences **(C)**.

For predicted plant PrLDs, pCARs were identified with Waltz at the three designated thresholds 73.5, 80, and 85. As expected, the percentage of PrLDs CAR positive (containing at least one CAR) increased in all species as the threshold became less stringent, hence allowing the detection of an increasing number of pCARs of polar nature (**Figure 2A**). Remarkably, at threshold 73.5, more than 90% of the predicted PrLDs contained such regions. According to the analysis, for the 5 tested plant species, PrLDs tend to display several pCARs, with and average length of >10 residues (**Figures 2B, C**). Comparing these metrics with generic intrinsically disordered regions (IDRs) extracted from DisProt (Santos et al., 2021), the positive rate of CARs in PrLDs is higher than in IDRs, as well as the number of CARs/LCD (low complexity domain, either PrLD or IDR) and their size. This indicates that CARs are more prevalent in PrLDs than in IDRs, likely because of the particular amino acid bias of prion LCD sequences.

As in IDRs (Santos et al., 2021), the widespread representation of CARs in plants is suggestive of a link with protein functionality. We compared the coincidence of pCARs from *A. thaliana* with linear interaction motifs (LIPs) as described in the MobiDB database (Piovesan et al., 2021), and observed a 63.5%, 65.6%, and 67.6% of pCAR residues overlapping with LIPs for the 73.5, 80 and 85 thresholds, respectively. This suggests that these regions might be conserved because of their contribution to establishing functional interactions. Our Aggrescan algorithm (Conchillo-Sole et al., 2007) failed to identify 71.5% (703/983) of *A. thaliana*s’ pCARs detected at the 80.0 threshold, indicating that, in the context of PrLDs, they do not confer to the containing protein a high risk for aggregation.

### 3.2 Amino acid composition of pCARs in *A. thaliana*

Once the presence of pCARs in all five plant species was confirmed and provided that the proportion of PrLDs and pCARs were similar, we selected the best-known plant model organism *A. thaliana* for subsequent analyses.

**Figure 3:**
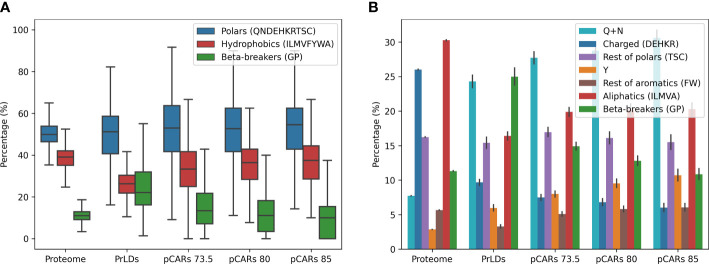
pCARs composition by threshold in *A thaliana* compared with their PrLDs and its proteome. Compared with the proteome, PrLDs already have an intrinsic bias toward polar and β-breakers amino acids. In contrast, pCARs are enriched in hydrophobics and depleted of β-breakers **(A)**. Most polar amino acids are QNs, and aromatic residues seem to modulate the amyloidogenic load of pCARs **(B)**. Barplot draws error bars in the plot with 95% confidence interval.

We compared the amino acid composition of PrLDs from *A. thaliana* with that of the complete proteome (**Figure 3A**). As expected from the encoded residue preferences in the employed PLAAC algorithm, in the PrLDs from *A. thaliana* are depleted of hydrophobic residues and enriched in β-breakers (GP), two features that play against the uncontrolled and generic aggregation of these disordered regions (Rousseau et al., 2006; Theillet et al., 2013). Indeed, glycines and prolines (G+P) alone equal the total content of hydrophobic residues, around 25%, whereas, in the total proteome, non-polar residues are threefold more abundant than G+P. Next, we compared pCARs composition against PrLDs. pCARs exhibit a higher percentage of hydrophobic residues and a lower content in β-breakers than PrLDs. As expected, the proportion of G+P in pCARs decreases as the Waltz threshold becomes more stringent. The percentage of hydrophilic residues in pCARs resembles that in PrLDs, being insensitive to the applied threshold, suggesting that they might provide background control to the equilibrium between disorder and assembly in these sequences.

To better dissect the relevant physicochemical features hidden in these amino acid groups, we split them into seven categories: Q+N, charged, rest of polars, tyrosine, rest of aromatics, aliphatics, and β-breakers. As observed in **Figure 3B**, only two residues (Q+N) account for almost 25% of the amino acids in PrLDs, a situation that contrasts with these residues’ low contribution to this plant proteome composition (7.9%). Asp and Gln are known to play a critical role in prion conformation (Perutz et al., 2002; Derkatch et al., 2004; Jiang et al., 2004; Sabate et al., 2015b; Zambrano et al., 2015; Zhang et al., 2016). Interestingly enough, Q+N are more frequent in pCARs than in complete PrLDs, their number increasing with the stringency of the prediction, indicative that they would be important contributors to the homo- or heterotypic interactions these stretches might establish. The higher proportion of hydrophobic amino acids in pCARs, relative to its PrLDs containers, can be traced to increases in both aromatic and aliphatic residues. However, it seems that the presence of aromatic residues is a distinctive feature of pCARs, their proportion increasing with the detection threshold. Specifically, tyrosines (Y) account in all the cases for more than 50% of the total aromatics, likely due to its dual polar/apolar character and its unique hydrogen bonding capability. This bias toward Y is reminiscent of what has been observed in liquid-liquid phase separation (LLPS), where phenylalanine and tyrosine are not interchangeable (Wang et al., 2018), likely because Y best balances solubility and assembly potential (Diaz-Caballero et al., 2018).

**Figure 4:**
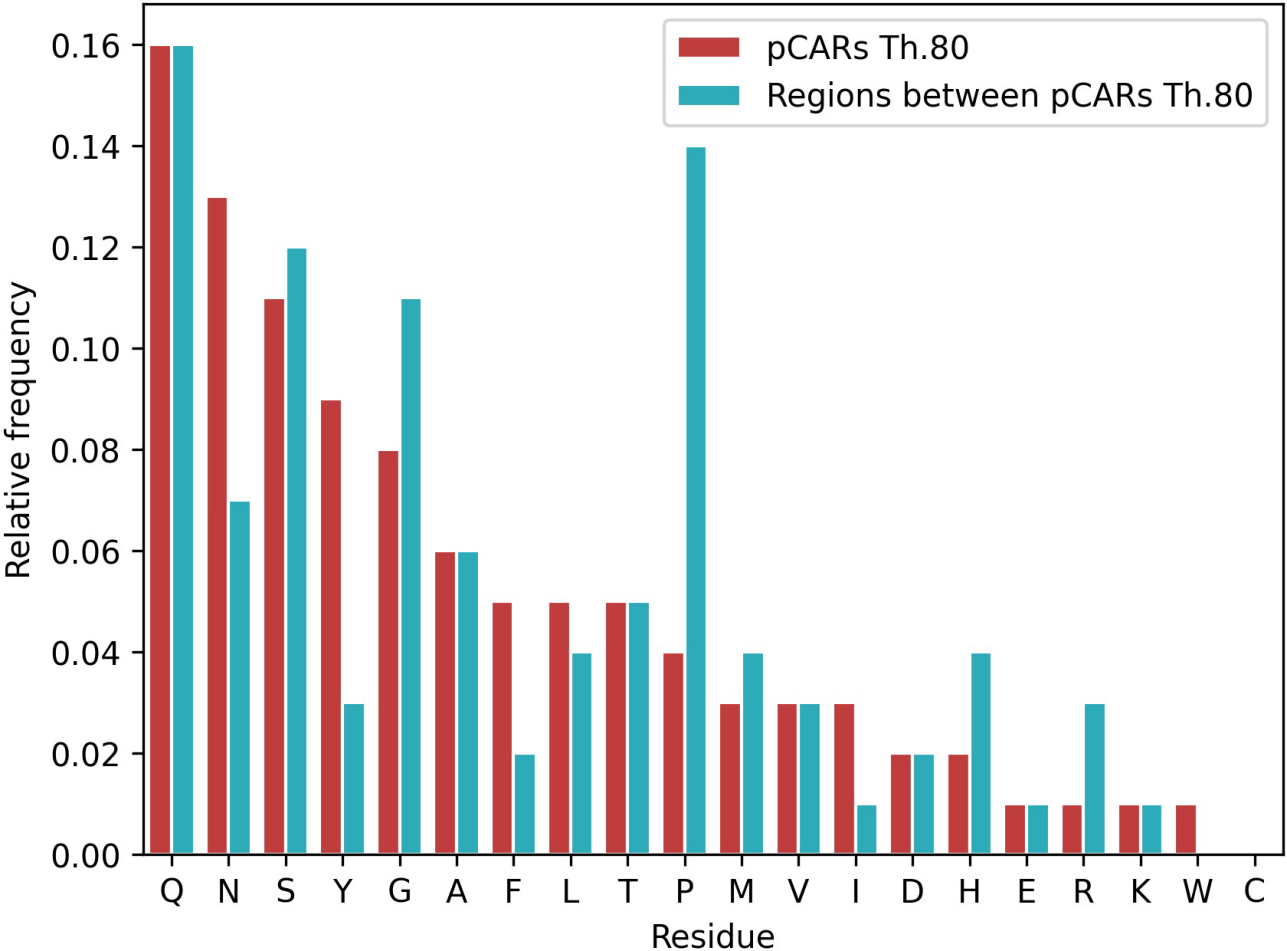
Residue composition comparison between *A. thaliana* pCARs and interpCARs (regions between two pCARs in any PrLD sequence) at threshold 80. pCARs are enriched in aromatic residues and depleted of β-breakers (GP). An opposite tendency is observed for interpCARs, which seem to compensate the sequential amyloidogenic load by reducing the aromatics and increasing β-breakers.

Not surprisingly, regions between pCARs (interpCARs) also differ in residues composition from pCARs (**Figure 4**). Compared with pCARs, interpCARs are highly enriched in G+P and depleted in aromatic residues. Despite Q+N are also abundant in interpCARs there is a strong preference for Q over N in these regions, indicating that their properties are not entirely exchangeable. Another interesting observation is that the cationic arginine (R) and histidine (H) residues are preferred at interpCARs, but this bias does not apply to other charged residues like lysine, glutamate, and aspartate. The average length of interpCARs is longer than that of pCARs; 20.4 and 12.7 at threshold 80.0, respectively.

In a way, the distribution and biased composition of pCARs within PrLDs is reminiscent of the proposed stickers and spacers model of LLPS (Wang et al., 2018; Martin et al., 2020; Holehouse et al., 2021; Bremer et al., 2022); with the Y+F+W+N enriched pCARs acting as stickers that facilitate protein interactions and the P+G+H+R enriched interpCARs as spacers, connecting stickers and providing them the adequate solubility and disorder context.

### 3.3 Functional enrichments of pCARs in *A. thaliana*

To explore the potential contribution of pCARs to the biological roles of *A. thaliana* prion-like proteins, we performed a functional enrichment analysis using the DAVID database (Sherman et al., 2022).

**Figure 5:**
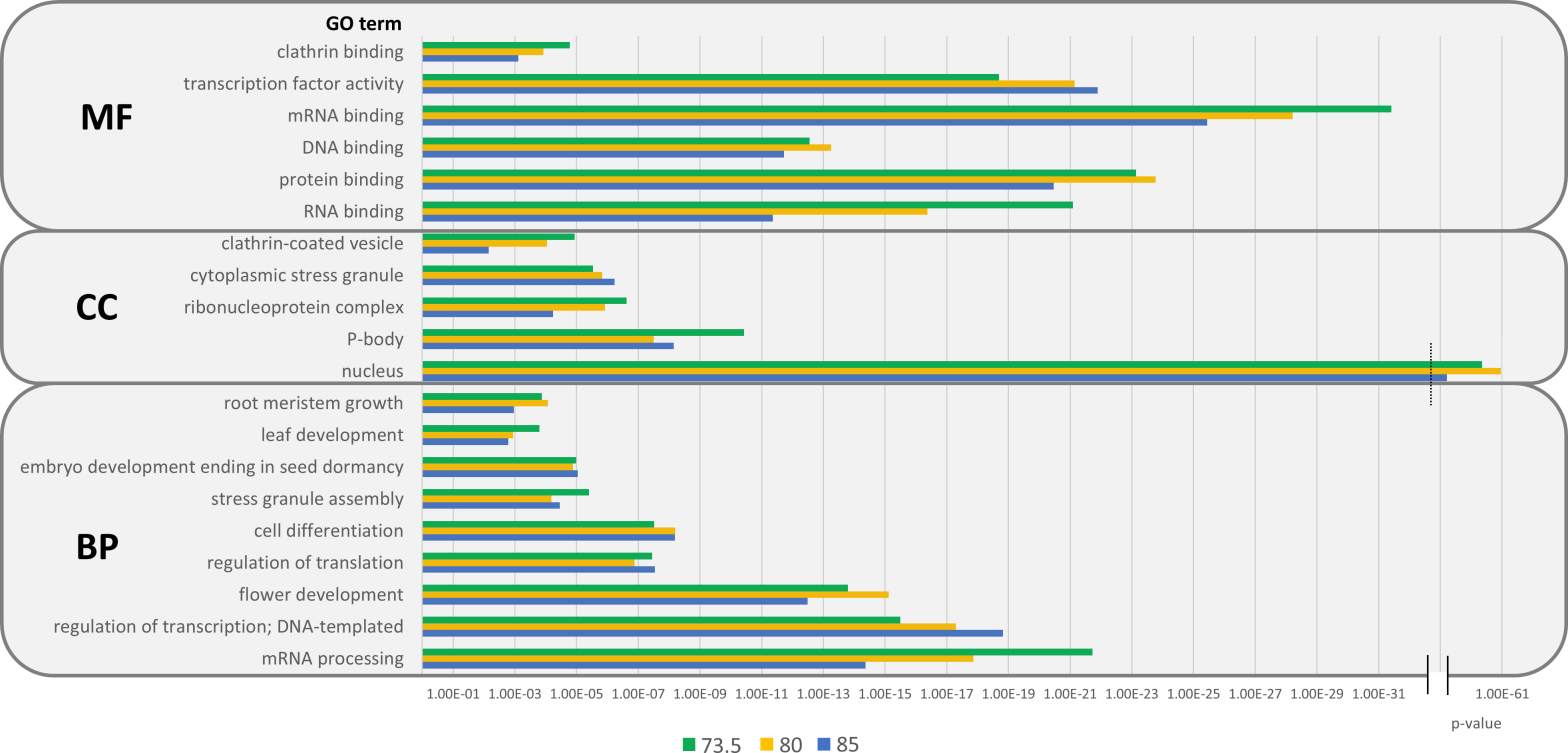
GO enrichments of pCARs from *A. thaliana* by threshold. Significant enrichments were found in cellular components (CC), biological processes (BP), and molecular functions (MF) at the three designated thresholds: 73.5 (green), 80 (yellow), and 85 (blue). pCARs are mainly associated with nuclear components and MLOs and are found in several regulatory processes and plant-specific functions.

Regarding the molecular functions of pCARs, significant enrichments were found for regulative processes, especially for nucleic acids, including DNA, RNA and mRNA binding, but also protein binding (**Figure 5**; **Supplementary Table 1**). This is interesting, as CARs have already been described to mediate key PPIs in IDPs (Santos et al., 2021; Pintado-Grima et al., 2022a). It is not surprising that, provided the previously proposed regulatory function of CARs, pCARs are found enriched in biological activities of gene expression, transcription, and translation. Related enrichments have been described in amyloidogenic (Antonets and Nizhnikov, 2017) and prion-like (Garai et al., 2021) proteins from plants. The fact that a significant proportion of PrLDs is pCAR-positive makes it challenging to disentangle the contributions of the complete PrLD sequence and pCARs only in a given protein to the detected enrichments. However, even when the *A. thaliana* prionome is used as background for the GO analysis, significant enrichments in regulatory processes such as regulation of transcription (BP), transcription factor activity (MF), or nucleus (CC) were still observed (**Supplementary Figure 1**). *A. thaliana*’s pCARs are also enriched in plant-specific biological processes such as leaf and flower development or root meristem growth. In fact, there is already experimental evidence for plant PrLDs being involved in the autonomous flowering pathway, specifically for the luminidependens PrLD from *A. thaliana* (Chakrabortee et al., 2016).

pCARs are found to be associated with nuclear components, ribonuclear complexes, and nucleus, the latter with the lowest p-values observed (p<10^-61^) among all GO terms analyzed. Certain membraneless organelles (MLOs), such as cytoplasmatic stress granules or P-bodies were also enriched in pCARs. PrLDs are recruited to MLOs in response to different stimuli (Boncella et al., 2020; Iglesias et al., 2021), and they need to have some condensate driving element that facilitates the formation of liquid droplets. This would be especially relevant when the protein phase separates by itself, in the absence of additional co-factors (Pintado-Grima et al., 2022b); as described, pCARs might act as sticker regions facilitating PrLDs’ coalescence and the stabilization of these cellular compartments (Tsoi et al., 2021). In general, RNA and mRNA binding and processing are associated with pCARs. This might reflect a situation in which many of the protein constituents of MLOs are originally nuclear RNA binding proteins (Mann and Donnelly, 2021), with many of them bearing PrLDs (Harrison and Shorter, 2017; Gotor et al., 2020).

### 3.4 MED9 pCAR form amyloid fibrils

The concept of CARs has been gaining strength by means of the continuous experimental validation of predicted segments of hydrophilic nature that form amyloid fibrils *in vitro* (Santos et al., 2021; Pintado-Grima et al., 2022a). Our goal is to increase the body of evidence on the intrinsic amyloidogenic potential of these sequences, when they are disconnected from their interpCARs regions. Accordingly, we examined a new pCAR from *A. thaliana*’s MED9 protein (Q8RWA2) in this work.

MED9, the mediator of RNA polymerase II transcription subunit 9, is a nuclear protein that is part of the mediator complex. It acts as a coactivator involved in the transcriptional regulation of almost all RNA polymerase II-related genes. The N-terminal domain of MED9 (1-149) is predicted as a PrLD by PLAAC. Within this PrLD, an 18-residue pCAR comprising residues 77 to 94 (77-QYQQFQQQQHFIQQQQFQ-94) emerges as an interesting pCAR that we selected by its overall polar composition (67%), significant aromatic content (22%), low Waltz score (77.87), and for being undetectable by well-established aggregation and amyloid predicting algorithms like Aggrescan (Conchillo-Sole et al., 2007; De Groot et al., 2012), Tango (Fernandez-Escamilla et al., 2004) or ZipperDB (Thompson et al., 2006).

**Figure 6:**
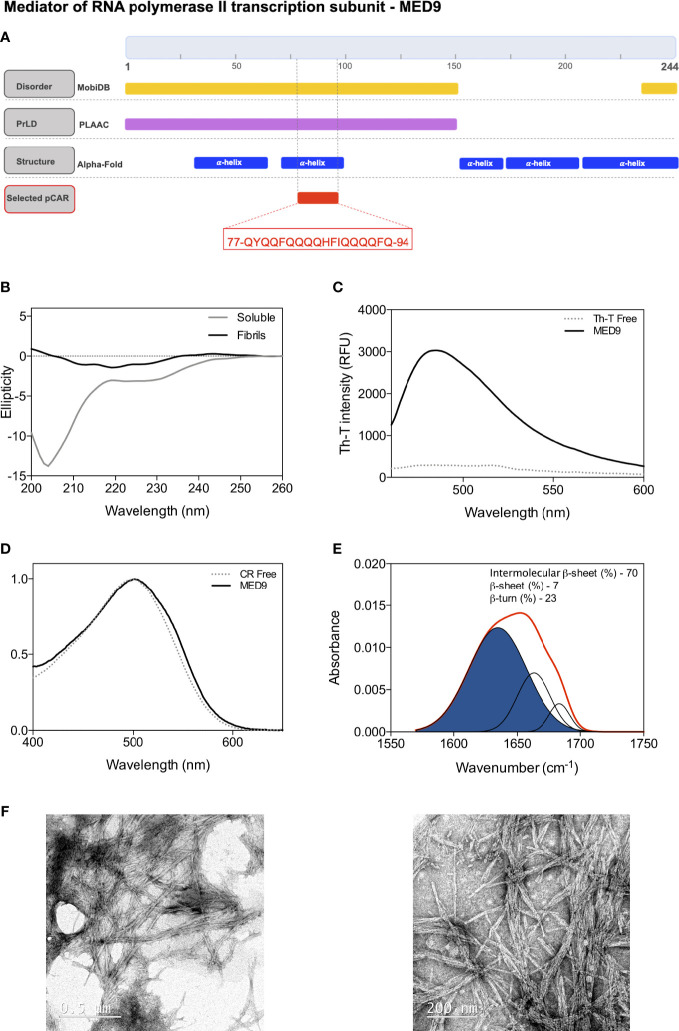
Experimental characterization of the predicted pCAR derived peptide in MED9 from *A thaliana*. **(A)** MED9 diagram showing the location of the predicted disordered regions (yellow), PrLD (violet), secondary structures (blue) and the selected pCAR (red). The sequence of pCAR is shown in the red box. **(B)** Far-UV circular dichroism spectra of the peptide, evolution from the initial disordered soluble state into mature amyloid fibrils at 50 μM and 48 hours of incubation at 37 °C with continuous agitation at 100 RPM. **(C)** Thioflavin-T fluorescence emission spectrum when excited at 440nm in the absence (dashed line) and presence (solid line) of the aggregated sample; note the characteristic fluorescence enhancement at 480 nm when the dye is bound to amyloid-like aggregates. **(D)** CR spectral changes in the absence (dashed line) and in the presence (solid line) of the incubated peptide. **(E)** FTIR spectrum in the amide I region of the incubated sample. The red line corresponds to the absorbance spectra and the blue area indicates the inter-molecular β-sheet contribution to the total area upon Gaussian deconvolution. **(F)** Representative TEM micrographs illustrating the fibrils of the incubated peptide.

MobiDB (Piovesan et al., 2021) consistently predicts MED9 PrLD to belong to a long-disordered region (1-150) (**Figure 6A**). AlphaFold (Jumper et al., 2021; Varadi et al., 2022) predict that residues 32-64 and 70-97 might form two alpha-helices, which, in the absence of tertiary contacts, are probably only attained upon binding to a molecular partner (i.e., coiled-coil formation). The MED9 pCAR maps within the second predicted helix (**Figure 6A**), suggesting a potential implication in PPIs through a folding upon binding mechanism, as reported in a significant proportion of previously characterized CARs (Santos et al., 2021).

To assess the amyloidogenic potential of MED9 pCAR, we analyzed whether a peptide corresponding to its sequence was able to self-assemble into amyloid fibrils *in vitro*. We incubated the peptide at 50 μM in 20 mM Tris and 100 mM NaCl pH 8 for 48 hours at 37 °C and 100RPM. After this time, the initial CD signal corresponding to the disordered soluble peptide was significantly reduced and shifted to a faint β-sheet signature, indicating protein aggregation (**Figure 6B**). The end-point sample was positive for amyloid dye binding (Th-T and CR), suggesting the formation of amyloid-like structures (**Figures 6C, D**). To assess the secondary structure content of the insoluble aggregates not accessible by CD, we used FTIR and recorded the amine I region of the infrared spectra (1700-1600 cm^-1^). We observed a dominant contribution of the 1630 cm^-1^ band (70% of the area) associated with the formation of amyloid-like intermolecular β-sheets (**Figure 6E**). A morphological analysis of the aggregates by TEM confirmed the fibrillar nature of the aggregates (**Figure 6F**).

Together, this data confirms that the MED9 pCAR has the inherent potential to aggregate into amyloid fibrils. Furthermore, the coincidence of predicted coiled-coil and amyloid propensities in the same sequence has been previously observed in a large number of human PrLDs and yeast prions (Fiumara et al., 2010; Batlle et al., 2021). Indeed, a human member of the mediator complex, MED15, is a *bona fide* prion-like protein whose PrLD transits between disordered, coiled-coil, and amyloid states (Batlle et al., 2021). As mentioned above, the MED9 pCAR is just one example of a conjunct of amyloid-competent sequences not detectable by classical aggregation predictors like our own Aggrescan (Conchillo-Sole et al., 2007).

## 4 Discussion

The prion phenomenon continues to attract high interest. In recent times, significant effort has been devoted to understanding the role PrLDs play in biological pathways across species, beyond those initially described in yeast prions. Although PrLDs seem to share a moderate amyloid propensity (Sabate et al., 2015b), the containing proteins do not always behave as *bona fide* prions, but rather exploit these LCDs to promote the functional interactions needed for their activity. Amyloid regions in PrLDs need to be milder and more polar than the hydrophobic stretches often identified in the core of pathogenic amyloid fibrils; to keep the protein soluble but allow specific inter-molecular interactions. In this scenario, the concept of CARs, whose existence has already been validated in generic IDPs (Santos et al., 2021), is translated here to PrLDs, since these domains are, by definition, intrinsically disordered. In the PrLD context, pCARs would define the boundaries of the regions that would concentrate this mild amyloid potential, flanked by longer and highly soluble interpCARs segments, acting analogously to the way stickers and spacers do in LLPS (Martin et al., 2020).

pCAR composition suggests that these regions within PrLDs arise from a reduction of β-breakers and an increase in hydrophobic residues, especially of an aromatic character, relative to the rest of the domain. In addition, Tyr seems to play a unique role in pCARs, since it units aromaticity and polarity, allowing interactions with water in the soluble state and pi-pi interactions in the assembled state. Notably, the content in polar residues of pCARs is equal to or higher than that of the complete PrLDs or even the whole proteome, which ensures that their amyloid propensity is framed in an overall soluble background. In this context, the polar Gln and specially Asn residues, which are highly enriched in PrLDs relative to the proteome but also in pCARs relative to these domains, would also contribute to control the amyloid load, since their side chains have a high tendency to form hydrogen bonds with the solvent and thus enable solubility, but at the same time, they can form very tight interactions within the dehydrated interfaces of β-strands (Balbirnie et al., 2001; Peccati et al., 2020). The preference for Asn relative to Gln in pCARs seems to make sense since Asn richness promotes the formation of benign assemblies, whereas Gln richness promotes the formation of toxic aggregates (Halfmann et al., 2011).

The present analysis uncovered a previously unexplored putative amyloid sequence space in plants PrLDs, relative to previous studies, including ours; where amyloid-prone regions identification was limited by rationally implemented but arbitrary requirements, including a fixed size, the absence of Pro or the belief that a single region would suffice to nucleate aggregation (Sabate et al., 2015a; Batlle et al., 2017b; Fernandez et al., 2017), essentially because the bias toward studying disease-associated amyloids made us wrongly thought that highly hydrophilic sequences, like pCARs, could not form amyloid structures autonomously. Importantly, the presence of these cryptic amyloidogenic regions seems to relate to RNA- and mRNA-related processes occurring in the nucleus, highlighting the significant role that PrLDs containing CARs might play in regulating plant-specific biochemical pathways.

Although the pCAR concept indeed requires extensive experimental validation before their presence can be established as a general principle modulating the activity of prion-like proteins, it is also certain that for most of the experimentally validated CARs, structural analysis indicated that these initially disordered regions might fold upon interaction with a partner, or, alternatively, that they mediate multimerization. This suggests that its higher amyloidogenic load, relative to the rest of the PrLD sequence, cannot be purged out by natural selection because CARs are needed for functional interactions. Surprisingly, in many instances, the folded structures adopted by CARs in functional complexes correspond to α-helices, often involved in coiled-coil dimerization (Batlle et al., 2021). Chameleon sequences adopt an α-helix in the folded state of globular proteins but display a hidden propensity to form β-sheets when unfolded, and α-helix/β-strand-discordant stretches were proposed to be associated with amyloid fibril formation already in 2001 (Kallberg et al., 2001). What makes special CARs, such as those of *A. thaliana* MED9 or human MED15, is that they might populate three different states, disordered in isolation, α-helix in functional PPIs, and β-sheets if they eventually transition to the amyloid state; with the three propensities encoded in the same sequence but controlled by the protein microenvironment and the sequential context.

We believe the approach described in the present study may translate to the detection of pCARs in other interesting prionomes, to build a comprehensive repository of polar amyloidogenic regions for further experimental validation and benchmarking future predictive algorithms.

## Data availability statement

The datasets presented in this study can be found in online repositories. The names of the repository/repositories and accession number(s) can be found in the article/**Supplementary Material**. The datasets are also available online at: http://carsdb.ppmclab.com/prionplants.

## Author contributions

CP-G, JS, and VI generated the dataset. CP-G and VI performed the analyses. ZM-A conducted the experimental validation. CP-G, IP, and SV conceptualized the project. CP-G, JS, and SV wrote the manuscript with the contributions of all authors. SV and IP acquired funding and reviewed the final version of the manuscript. All authors have read and agreed to the published version of the manuscript.
