# Supplementary material for: Exploring cryptic amyloidogenic regions in prion-like proteins from plants
Source: Front Plant Sci. 2023 Jan 16;13:1060410. doi: 10.3389/fpls.2022.1060410 (PMC9885169; doi:10.3389/fpls.2022.1060410)
Supplement: Supplementary file 1 [file DataSheet_1.zip › Supplementary_Figures.pdf]

Supplementary material for:

Exploring Cryptic Amyloidogenic Regions in Prion-like Proteins from Plants

Carlos Pintado-Grima, Jaime Santos†, Valentín Iglesias†, Zoe Manglano-Artuñedo, Irantzu Pallarès and Salvador Ventura[1*]

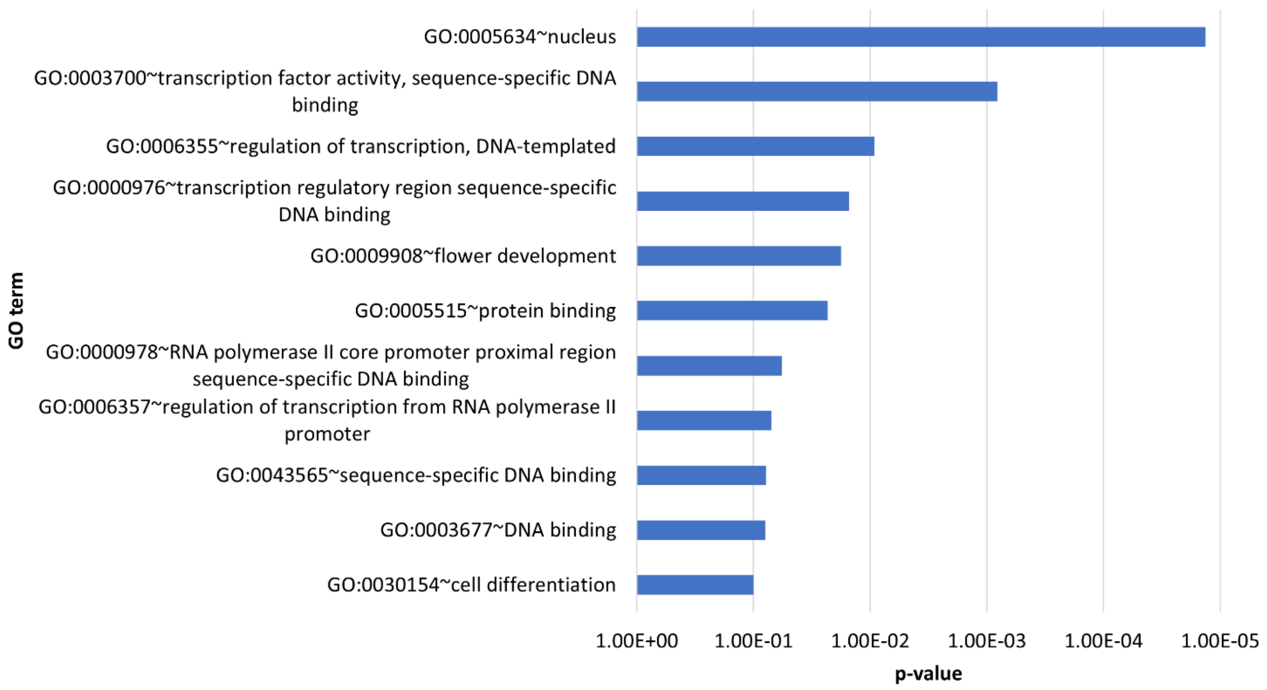

Supplementary Figure 1. GO enrichments obtained for *A. thaliana* pCARs at threshold 80 using its PrLDs as background. Despite most of *A. thaliana* PrLDs are CAR positive, when its prionome is used, significant pCARs enrichments are still observed for several regulatory processes.
